# Screening Phosphorylation Site Mutations in Yeast Acetyl-CoA Carboxylase Using Malonyl-CoA Sensor to Improve Malonyl-CoA-Derived Product

**DOI:** 10.3389/fmicb.2018.00047

**Published:** 2018-01-25

**Authors:** Xiaoxu Chen, Xiaoyu Yang, Yu Shen, Jin Hou, Xiaoming Bao

**Affiliations:** ^1^State Key Laboratory of Microbial Technology, School of Life Science, Shandong University, Jinan, China; ^2^Shandong Provincial Key Laboratory of Microbial Engineering, Qilu University of Technology, Jinan, China

**Keywords:** malonyl-CoA sensor, phosphorylation site mutations, acetyl-CoA carboxylase, 3-hydroxypropionic acid, *Saccharomyces cerevisiae*

## Abstract

Malonyl-coenzyme A (malonyl-CoA) is a critical precursor for the biosynthesis of a variety of biochemicals. It is synthesized by the catalysis of acetyl-CoA carboxylase (Acc1p), which was demonstrated to be deactivated by the phosphorylation of Snf1 protein kinase in yeast. In this study, we designed a synthetic malonyl-CoA biosensor and used it to screen phosphorylation site mutations of Acc1p in *Saccharomyces cerevisiae*. Thirteen phosphorylation sites were mutated, and a combination of three site mutations in Acc1p, S686A, S659A, and S1157A, was found to increase malonyl-CoA availability. *ACC1^S686AS659AS1157A^* expression also improved the production of 3-hydroxypropionic acid, a malonyl-CoA-derived chemical, compared to both wild type and the previously reported *ACC1^S659AS1157A^* mutation. This mutation will also be beneficial for other malonyl-CoA-derived products.

## Introduction

*Saccharomyces cerevisiae* is a very potential microbial cell factory for production of a wide range of fuels and chemicals due to its robustness and high tolerance to environmental stresses. When engineering the cell factory to produce these products, the precursor availability is one of key limiting factors that affects the product titer. Malonyl-coenzyme A (malonyl-CoA) is a major precursor for the production of many important biochemicals and biofuels, such as polyketides, flavonoids, and fatty acid-derived chemicals (Dixon and Steele, 1999; Hara et al., 2006; Christian, 2009; Chooi and Tang, 2013). However, the intracellular level of malonyl-CoA is very low because malonyl-CoA synthesis is tightly regulated in *S. cerevisiae* (Li et al., 2014). Therefore, insufficient malonyl-CoA synthesis largely limits the production of malonyl-CoA-derived products.

In *S. cerevisiae*, cytosolic malonyl-CoA is synthesized from acetyl-CoA by acetyl-CoA carboxylase (Acc1) and it is subsequently used for fatty acid synthesis. Acc1p is crucial for fatty acids biosynthesis and the maintenance of the nuclear envelope. Acetyl-CoA carboxylases are large, multidomain enzymes and contain a biotin carboxylase (BC) domain, a carboxyltransferase (CT) domain and a biotin carboxyl carrier protein (BCCP) domain. Eukaryotic ACCs also contain two non-catalytic regions, the large central domain (CD) and the BC–CT interaction domain (BT) (Hunkeler et al., 2016). As the rate-limiting enzyme for fatty acid metabolism, Acc1p is tightly regulated both transcriptionally and post-translationally. Similar to other phospholipid synthesis pathway genes, the transcription of *ACC1* is regulated by the positive transcription factors Ino2p and Ino4p and the negative regulator Opi1p (Hasslacher et al., 1993). The enzyme activity of Acc1p is also negatively regulated by AMP-activated protein kinase (Snf1) in *S. cerevisiae* (Woods et al., 1994). It is reported that the phosphorylation of Acc1p by Snf1p inactivates the catalytic ability of Acc1p. The activation of Snf1p is triggered during growth under glucose derepression conditions (less than 0.05% glucose) (Woods et al., 1994), then the activated Snf1p phosphorylates Acc1p at one or more phosphorylation sites and results in the partial deactivation of Acc1p. However, potential residues for Acc1p phosphorylation are not fully understood. Feng et al. (2015) reported that deletion of Snf1 did not increase the 1-hexadecanol production, but overexpressing *ACC1* in *ΔSNF1* strain increased the production by 50% (Feng et al., 2015). Asides from regulating *ACC1*, Snf1 is also a global regulator for carbon metabolism, β-oxidation, and stress response (Zhang et al., 2011). Thus, *SNF1* deletion may not specifically increase malonyl-CoA derived products.

Choi and Da Silva (2014) aligned the *Rattus norvegicus* (rat) Acc1p with yeast Acc1p and identified an amino acid (Ser1157) that is critical for phosphorylation and causing deactivation of the enzyme. The introduction of a S1157A mutation in Acc1p increased its activity and enhanced polyketide 6-methylsalicylic acid and native fatty acid production (Choi and Da Silva, 2014). A recent report demonstrated that the phosphorylation of the Ser1157 residue by Snf1p triggered a conformational change, leading to a disassociated BC domain dimer, thus inactivating the catalytic ability of Acc1p (Wei et al., 2016). Shi et al. identified two phosphorylation sites that were regulated by Snf1p by comparing the phosphorylation recognition motif (Hyd-X-Arg-XX-Ser-XXX-Hyd) (Dale et al., 1995) and introduced a two-site mutation (S659A and S1157A) in Acc1p. These mutations enhanced the activity of Acc1p and subsequently increased the production of two malonyl-CoA-derived products, fatty acid ethyl esters and 3-hydroxypropionic acid (3-HP) (Shi et al., 2014). However, although these studies reported the positive role of mutations S659A and S1157A, it is still uncertain whether Snf1p regulates Acc1p only by phosphorylating these two sites. Li et al. (2014) manually examined the modification sites on Acc1p using MIDAS and confirmed that 15 sites were phosphorylated. The authors also noted that both the native state and phosphorylated state were identified at the same time, indicating that the regulation of phosphorylation is complicated.

The Acc catalytic activity determination is complicated, therefore limited the high-throughput screening the mutation libraries of the enzyme. Intracellular malonyl-CoA concentration is another way to reflect the Acc activity. The malonyl-CoA biosensor has been designed in *S. cerevisiae* to facilitate the malonyl-CoA determination using a bacterial transcription factor FapR (fatty acid and phospholipid biosynthesis regulator) and its DNA binding site (*fapO* operator) (Schujman et al., 2003). Li et al. (2015) designed a malonyl-CoA sensor by inserting a *fapO* operator immediately upstream the TATA box in *GPM1* promoter and expression of an adapted FapR to monitor the malonyl-CoA levels in *S. cerevisiae*. By using this sensor to search a genome-wide overexpression library, two genes, *TPI1* and *PMP1*, were identified that improved the intracellular malonyl-CoA concentration, thereby improving 3-HP production (Li et al., 2015). David introduced three *fapO* operators in yeast *TEF1* promoter to increase the sensitivity of responding the malonyl-CoA concentration and used it to modulate pathway flux. The introduction of dual pathway control increased 3-HP production (David et al., 2016). However, these sensors either have a relatively high leaking transcription or low dynamic change response to malonyl-CoA levels (**Table 1**).

**Table 1:** Designs and characters of the malonyl-CoA sensors in *S. cerevisiae*.

Promoter used in sensor	Numbers of *FapO* operator	Level of FapR	The fold change of fluorescence intensity (with FapR/without FapR)	The fold change of fluorescence intensity (with saturated cerulenin/without cerulenin)	Reference
*GPM1*	1	High	29.8%	4	Li et al., 2015
*TEF1*	3	Low	14%	1.9	David et al., 2016
*TEF1* UAS and *GAL1* core	1	High	0.05%	8	This study

**FIGURE 1:**
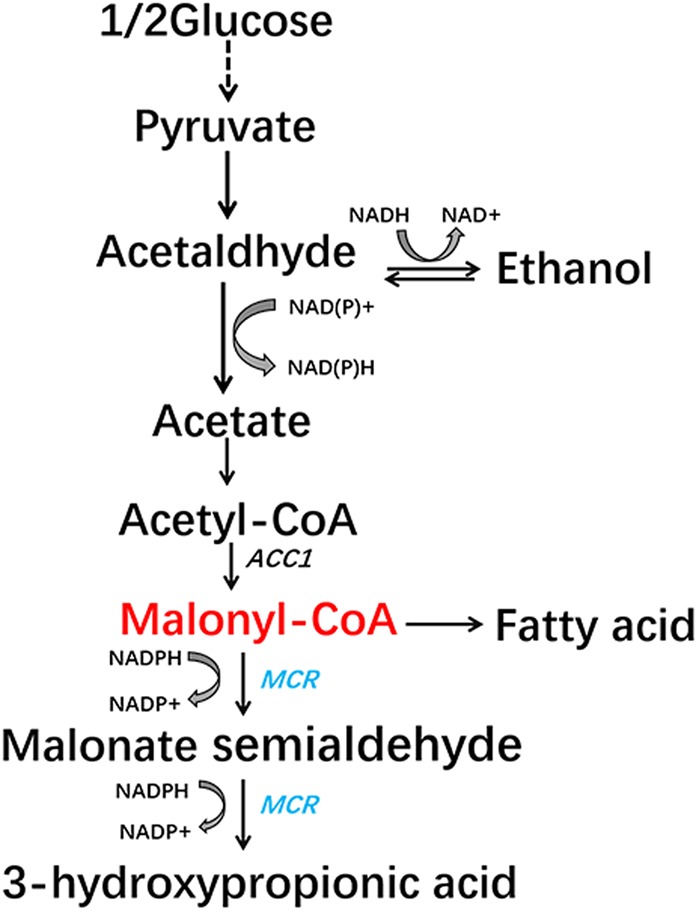
Malonyl-CoA biosynthesis metabolic pathway and the engineered 3-hydroxypropionic acid synthesis pathway in *S. cerevisiae*.

In this study, we aimed to construct a malonyl-CoA sensor in *S. cerevisiae* that has higher sensitivity and low leaky transcription which is very important for library screening. The sensor we designed showed better response to malonyl-CoA concentrations than previously reported sensors. Then we used the sensor to detect the effect of mutating all possible phosphorylation sites of Acc1p. A mutation, Acc1p (S686AS659AS1157A), that could increase the concentration of malonyl-CoA and the production of 3-HP (a chemical derived from malonyl-CoA) (**Figure 1**), was isolated.

## Materials and Methods

### Medium and Growth Conditions

*Saccharomyces cerevisiae* strain CEN.PK102-5B (*MATa; ura3-52; his3Δ1; leu2-3,112*) was used as the background strain. Synthetic complete (SC) dropout medium was used for recombinant yeast strain selection. The SC medium contained 1.7 g/L yeast nitrogen base (BBI Life Science Corporation, China), 5 g/L ammonium sulfate, complete supplement mixture (without uracil, histidine, or leucine) (Sunrise Science Products, United States), and 20 g/L glucose. In total, 800 μg/mL G418 (Promega Corporation, United States) was used for selecting strains with the *Kan^r^* marker. For routine cloning procedures, *Escherichia coli* Trans2-Blue (Beijing TransGen Biotech Co., Ltd, China) was used and grown in Luria–Bertani (LB) medium with 100 μg/L ampicillin.

### Plasmid and Strain Construction

All the plasmids and strains used in this study are listed in Supplementary Tables S1 and S2. The primers used in this study are listed in Supplementary Table S3. Yeast strain transformation was accomplished using the LiAc method. The obtained plasmids were verified by DNA sequencing.

The malonyl-CoA reductase encoding gene *MCR* from *Chloroflexus aurantiacus* was codon optimized, digested with *Not*I and *Sac*I, and ligated into the 2 μ plasmid pIYC04 under the control of the *TEF1* promoter and *ADH1* terminator. T4 DNA ligase (New England Biolabs, United States) was used for plasmid construction.

**FIGURE 2:**
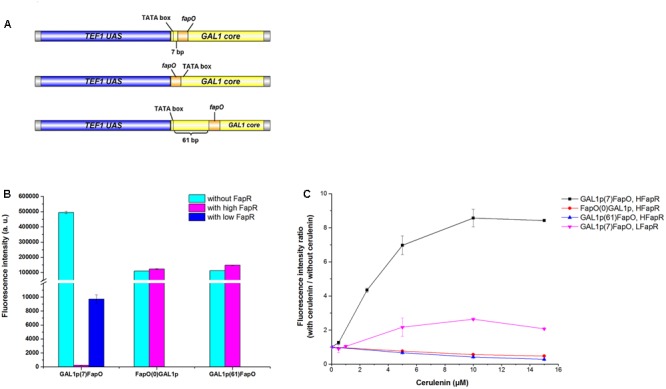
Design and characters of the synthetic malonyl-CoA sensors. **(A)** Design of the synthetic promoters. The positions of *fapO* sequence were as follows: 7 bp downstream the TATA box of *GAL1* core promoter, immediately upstream the TATA box and 61 bp downstream the TATA box of *GAL1* core promoter. **(B)** Comparing of the fluorescence intensity of the sensors between with FapR and without FapR. The synthetic promoters were cloned in multiple-copy plasmid. Fluorescence intensity (a.u.) means total fluorescence normalized by cell density (OD600). **(C)** The fluorescence intensity fold change of the sensors with gradient concentration of cerulenin addition relative to zero cerulenin addition. HFapR: *fapR* was expressed in a multicopy plasmid; LFapR: *fapR* was integrated into genome.

For a high level of *fapR* expression, the codon-optimized *fapR* was digested with *Sac*I and *Sal*I and then ligated into the pre-digested 2 μ plasmid pYX242WS, resulting in the plasmid pYX242WS-fapR. A synthetic promoter was constructed by combining 203 bp of the *TEF1* promoter upstream activation sequence and 147 bp of the *GAL1* core promoter (with an identified TATA box). The FapR binding site, 17 bp of the *fapO* operator (TTAGTATCAGGTACTAA), was inserted into the *GAL1* core promoter, 7 bp immediately behind the TATA box and replaced the original sequence to obtain the synthetic promoter GAL1p(7)fapO (**Figure 2A**) (Schujman et al., 2003; Blazeck et al., 2012; Xu et al., 2014). The *yeGFP* (yeast-enhanced green fluorescent protein-encoding gene) was fused with the synthetic promoter by fusion PCR, and the fragment was then digested with *Aat*II and *Eco*RI and inserted into the pre-digested pYX242WS or pYX242WS-fapR plasmid to create pJfapO and pJfapO-fapR. To obtain synthetic promoters GAL1p(61)fapO and fapO(0)GAL1p, the *fapO* operator was inserted 61 bp behind or immediately upstream the TATA box separately. Each of the synthetic promoters was fused with *yeGFP* and then was cloned into a 2 μ plasmid to obtain plasmid pIFapO(0) and pIFapO(61) through yeast homologous recombination. The codon-optimized *fapR* with SV40 NLS sequence at the 3′ end was digested with *Not*I and *Sac*I and then cloned into plasmids pIFapO(0) and pIFapO(61) separately to create pIFapO(0)-FapR and pIFapO(61)-FapR. To obtain a low level of *fapR* expression, *fapR*, which was controlled by the *TEF1* promoter, was integrated into the yeast genome using *Kan^r^* as the selection marker. The plasmid pJFE3-fapO was transformed into the *fapR* integration strain. The sequence information of *fapR* and *MCR* was included in supplemental data.

Site-directed mutagenesis was introduced by designing mutant sites containing primers. A full *ACC1* sequence with mutated residues was obtained by fusion PCR. *ACC1* mutations were inserted into the 2 μ plasmid pJFE3 under the control of the *TEF1* promoter and *PGK1* terminator through yeast homologous recombination. The constructed plasmids were verified by PCR and sequencing.

### Malonyl-CoA Sensor Activity and Acc1p Mutation Assay

Fluorescence intensity was used to evaluate the sensor activity. The sensor-carrying strains were pre-cultured overnight. The overnight culture was collected and inoculated into 3 mL of fresh SC medium at an initial OD600 of 1 at 30°C, 200 rpm, and different concentrations of cerulenin (0, 0.5, 2.5, 5, 10, 15 μM) were added if necessary. For the fluorescence intensity assay, a 1 mL culture was isolated after 2 h of growth and centrifuged at 4°C and 13,000 rpm for 2 min, and the pellets were washed twice with distilled water. A 200 μL cell suspension was used for fluorescence intensity measurement in triplicate. Fluorescence was measured using a 1420 Multilabel Counter (VICTOR^3^V, PerkinElmer, United States). The excitation and emission wavelengths for GFP were 485 ± 20 and 585 ± 20, respectively. The cell density was measured at 600 nm (Eppendorf BioPhotometer, Germany). The fluorescence intensity (a.u.) was determined relative to cell density. To detect the fluorescence intensity of the mutations, the strains were pre-cultured overnight, inoculated in shake flasks at an initial OD600 of 0.2 in 10 mL of SC medium, and cultivated 30°C at 200 rpm. Samples were taken after 6 h, and fluorescence was measured as described above.

### 3-HP Fermentation

For 3-HP fermentation, strains were pre-cultured in 5 mL of SC dropout medium, and the seed culture was transferred into a 100 mL shake flask at a working volume of 40 mL. The initial OD600 of the culture was 0.2. Fermentation was performed at 200 rpm and 30°C. Samples were taken every 12 h until 72 h. 3-HP was analyzed by HPLC (Shimadzu Corporation, Japan) equipped with an Aminex HPX-87H column (Bio-Rad, Hercules, United States) at 65°C using 2.5 mM H_2_SO_4_ as the mobile phase at a flow rate of 0.6 mL/min.

## Results

### Design an Efficient Malonyl-CoA Sensor in *S. cerevisiae*

Malonyl-CoA acts as a direct and specific inducer of FapR-regulated promoters in *Bacillus subtilis* (Schujman et al., 2006). FapR binds to *fapO* (the operator region on the *fap* regulon) in the promoter region, which blocks the access of RNA polymerase II, thus repressing the transcription of downstream fatty acid pathway genes. Malonyl-CoA interacts with FapR and triggers a conformational change in FapR, consequently enabling FapR to disassociate from DNA and relieve the transcription repression. Based on that, we designed a malonyl-CoA sensor in *S. cerevisiae* and used *yeGFP* as a reporter to monitor the intracellular malonyl-CoA levels. To obtain a more efficient sensor, the leaking transcription should be low, which means that when fapR binding to *fapO*, the basic transcription level should be low. In addition, the sensor also needs to response to the change of malonyl-CoA level sensitively, which means that the dynamic change of the fluorescence intensity should be high. Therefore, we chose a synthetic hybrid promoter containing the upstream activated sequence (UAS) of *TEF1* promoter and core promoter sequence (begin with TATA box) of *GAL1*. The UAS from *TEF1* allowed the high expression of downstream gene without fapR binding, and TATA box in the core promoter facilitate to decide the locations of *fapO* operator. The FapR binding site, 17 bp of the *fapO* operator, was inserted either 7 bp or 61 bp downstream of the TATA box in *GAL1* core promoter, or immediately upstream the TATA box, and *yeGFP* was controlled by these synthetic hybrid promoters in a 2 μ plasmid (**Figure 2A**). The codon-optimized *fapR* was expressed under the control of the *TEF1* promoter in a 2 μ plasmid. When *fapO* was inserted 7 bp downstream of the TATA box [GAL1p(7)fapO for briefness], the transcription was almost completely shut down. The fluorescence intensity with FapR was only 0.05% relative to that without FapR, demonstrating the efficient repression effect of FapR when binding to this position (**Figure 2B**). However, when *fapO* was inserted 61 bp downstream of the TATA box [GAL1p(61)fapO for briefness] or immediately upstream of the TATA box [fapO(0)GAL1p for briefness], no transcription repression was observed, demonstrating that the location of *fapO* on these two sites was not efficient enough for FapR to inhibit transcription initiation.

Besides the effect of *fapO* location in promoter, we also detected the expression level of FapR to the behavior of the sensor. The *fapR* was integrated into the genome to obtain low expression level, and the *yeGFP* was controlled by the GAL1p(7)fapO synthetic promoter. We found that compared with the FapR expressed in a 2 μ plasmid, a low level of FapR expression resulted in high fluorescence intensity which implicated that low FapR expression caused high leaky transcription (**Figure 2B**).

Cerulenin inhibits fatty acid synthesis by specific inhibition of β-ketoacyl-acyl carrier protein synthetase (Kawaguchi et al., 1982), and inhibits malonyl-CoA consumption for fatty acid synthesis. Thus, the intracellular malonyl-CoA concentration correlates with the amount of cerulenin added to the culture. As shown in **Figure 2C**, as for the sensor containing the GAL1p(7)fapO synthetic promoter, with a gradient supply of cerulenin, the fluorescence intensity also increased gradually, reaching to eightfold with 10 μM cerulenin (saturated concentration) relative to the condition without cerulenin in high FapR expression strain. The fluorescence intensity only increased onefold with cerulenin supply in low FapR expression strain (**Figure 2C**). As expected, the fluorescence intensity of the sensor containing fapO(0)GAL1p or GAL1p(61)fapO synthetic promoter did not increase with increased dose of cerulenin addition, indicating these two designs could not respond the malonyl-CoA concentration. These results demonstrated that high FapR expression combined with GAL1p(7)fapO synthetic promoter showed better response to the change of malonyl-CoA levels.

Comparing with the previously reported malonyl-CoA sensor (**Table 1**), the sensor with GAL1p(7)fapO synthetic promoter and high FapR expression designed in this study showed a low leaky transcription and a better sensitivity to the change of malonyl-CoA levels, which are very important properties for library screening.

### Site-Directed Mutations of Phosphorylation Sites of Acc1p Increased Intracellular Malonyl-CoA Levels

**FIGURE 3:**
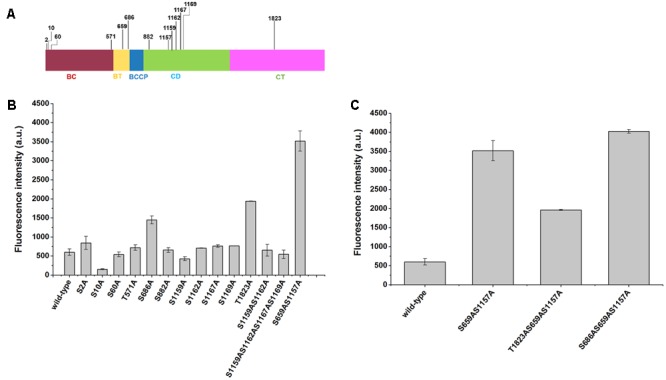
Positions of the phosphorylation sites in Acc1p and the fluorescence intensity of phosphorylation mutation strains containing the malonyl-CoA sensor. **(A)** Position of the 13 phosphorylation sites in our study. **(B)** Fluorescence intensity at 6 h of the strains. Wild-type: CEN.PK102-5B with an empty plasmid and malonyl-CoA sensor plasmid. **(C)** Fluorescence intensity of the strains with combined mutations. The fluorescence intensity was measured at exponential phase.

Although mutations of Ser659 and Ser1157 phosphorylation sites have been shown to improve the synthesis of malonyl-CoA-derived products, it remains unclear whether other phosphorylation sites may have a positive impact. In this work, we mutated 13 predicted phosphorylation sites of Acc1p according to Li et al.’s (2014) report (**Figure 3A**), which confirmed 15 phosphorylation sites of Acc1p via MIDAS. The other two predicted phosphorylation residues are not existed in the Acc1p from CEN.PK background strain used in this work. The phosphorylation residues were mutated to alanine. The sensor plasmid was then transformed into the Acc1p mutation-containing strains to screen the malonyl-CoA-overproducing mutants. We found that the fluorescence intensity of strains containing Acc1p(S686A) and Acc1p(T1823A) was increased relative to the wild type, even though the increased range was still lower than strain with the previously reported Acc1p(S659AS1157A) double mutation. The remaining phosphorylation site mutations did not show a positive effect on malonyl-CoA levels (**Figure 3B**). We further combined S686A and T1823A single mutations with the S659AS1157A double mutation to see if the combination mutations can achieve further increase in malonyl-CoA generation. As shown in **Figure 3C**, the introduction of the S686A mutation further improved the fluorescence intensity of the *ACC1^S659AS1157A^*-expressing strain, whereas T1823A did not further enhance the fluorescence intensity, although the fluorescence intensity of the Acc1p(T1823A) mutant was higher than wild-type Acc1p. These results indicated that Acc1p(S686AS659AS1157A) can produce more malonyl-CoA and should have higher enzyme activity.

### The Effect of Site Mutations of Acc1p to 3-HP Production

**FIGURE 4:**
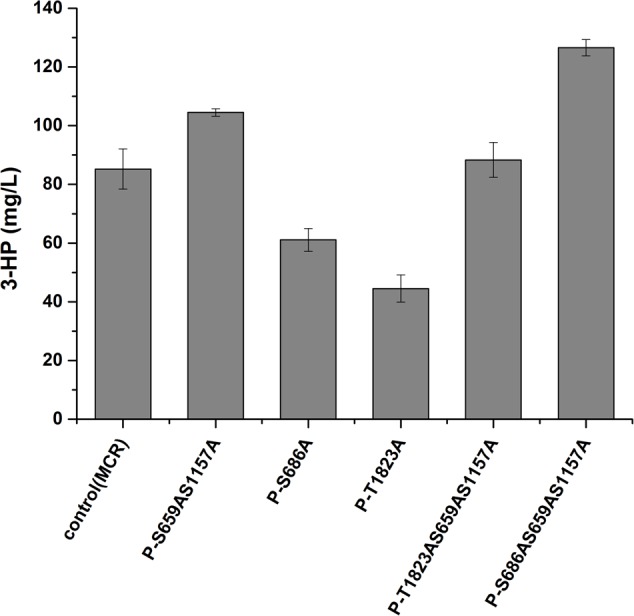
3-HP production by the strains expressing phosphorylation site mutations of Acc1p. Samples were taken at 72 h in shake flask fermentation. Control (MCR): CEN.PK102-5B with the *MCR*-containing plasmid.

We then analyzed the production of 3-HP, a malonyl-CoA-derived chemical, to determine whether the increase in malonyl-CoA would result in higher production of malonyl-CoA-derived chemical (**Figure 1**). The heterogeneous gene *MCR*, which encodes a bifunctional enzyme that includes malonyl-CoA reductase and 3-hydroxypropionate dehydrogenase, was overexpressed in Acc1p mutation strains (Hugler et al., 2002). The titer of 3-HP in the *ACC1^S659AS1157A^*-expressing strain was improved by 1.2-fold relative to the wild type, which is consistent with the positive role of the S659AS1157A mutation in a previous study (Shi et al., 2014). The titer of 3-HP in the *ACC1^S686AS659AS1157A^*-expressing strain was improved by 1.5-fold compared with the wild type, which is higher than the *ACC1^S659AS1157A^*-expressing strain (**Figure 4**). This finding confirmed that the S686AS659AS1157A mutation of Acc1p contributed to an increase in malonyl-CoA availability. However, the S686A single mutation of Acc1p did not improve 3-HP production. This result indicated that S686A could benefit 3-HP production only when integrated with the S659AS1157A mutation. Additionally, although the fluorescence intensity of *ACC1^T1823A^*- and *ACC1^T1823AS659AS1157A^*-expressing strains was higher than the wild type, 3-HP production did not increase, excluding the positive role of T1823A for 3-HP generation. Cell growth of the engineered strains was not obviously changed by *ACC1* mutation expression (Supplementary Figure S2).

## Discussion

The low production of malonyl-CoA in yeast constrains the biosynthesis of many valuable malonyl-CoA-derived compounds, such as flavonoids, polyketides, and fatty acid-derived fuels. Malonyl-CoA is generated from acetyl-CoA by the catalysis of acetyl-CoA carboxylase. The activity of Acc1p is tightly regulated, and the phosphorylation of Acc1p by Snf1 protein kinase can inactivate Acc1p. Mutations of the phosphorylation sites Ser1157 and Ser659 have been shown to enhance the activity of Acc1p and consequently the ability to synthesize malonyl-CoA-derived products (Choi and Da Silva, 2014). However, there are more phosphorylation sites in Acc1p. Li et al. (2014) predicted 15 possible phosphorylation sites in Acc1p by *in vitro* experiments, and it is necessary to explore the role of these phosphorylation sites in the enzymatic bioactivity of acetyl-CoA carboxylase.

To facilitate the screening of phosphorylation site mutations, a malonyl-CoA sensor was constructed. Compared with the other two previously reported malonyl-CoA sensors (Li et al., 2015; David et al., 2016), the sensor we designed in this work showed low leaky transcription and better sensitivity to the change of malonyl-CoA concentrations (**Table 1**). Although these malonyl-CoA sensors used operators from the same origin (*fapO* from *B. subtilis*), the behavior is very different when inserting the operator to different promoters. As for the *TEF1-GAL1* synthetic promoter in this work, the transcription was not repressed when *fapO* was located immediately upstream TATA box, while the same position in *GPM1* promoter worked well. When the distance between *fapO* and the TATA box in *TEF1-GAL1* synthetic promoter extended to 61 bp, the transcription repression effect was disappeared. It seems that inserting one operator 7 bp downstream of TATA box in TATA-containing promoter is enough for FapR to repress the transcription initiation, whereas it is not the case when the operators were inserted to the TATA-less promoter *TEF1* where three operators were needed to repress the transcription effectively. In fact, the promoter property and operator locations are important to decide the performance of the biosensors.

Among the phosphorylation site mutations, the S659AS1157A double mutation increased 3-HP production, which confirmed the role of these phosphorylation sites. Combining the S686A mutation with the S659AS1157A double mutation further increased 3-HP production, suggesting that the phosphorylation site mutation of Ser686 further improved the enzyme activity of Acc1p. Ser1157 phosphorylation triggers a conformational change that disables the formation of the BC dimer domain, thus eliminating the catalysis activity (Hunkeler et al., 2016). However, there is no direct evidence regarding the inhibitory regulation induced by the phosphorylation of Ser659. By analyzing the Acc1p structure (from RCSB Protein Data Bank) using PyMOL software, we speculated that phosphorylation of Ser659 might influence the biotin moiety binding by regulating the active loop in BC domain. It is possible that the phosphorylation of Ser686 affected the enzyme activity through indirectly regulating the covalent state of Ser659 (Supplementary Figure S1).

We found that the fluorescence intensity was not completely consistent with 3-HP production. Only the strains with much higher fluorescence intensity showed a beneficial effect on 3-HP production. It is probably because malonyl-CoA is an intermediate metabolite and can be consumed easily. Synthetic malonyl-CoA sensor can show the transient malonyl-CoA concentration. However, the production of 3-HP revealed the amount of malonyl-CoA flux to the downstream products. Therefore, when using a malonyl-CoA sensor to do high-throughput screening, it is essential to set up a fluorescence intensity threshold to obtain the mutations with higher products titer.

In this work, we studied the impact of phosphorylation regulation on the activity of acetyl-CoA carboxylase. We discovered that a combination mutation with S686A, S659A, and S1157A could benefit 3-HP production better than the previously reported S659S1157A mutation. Moreover, the malonyl-CoA sensor designed in this work was demonstrated to be a useful tool to screen mutations with improved malonyl-CoA pools.

## Author Contributions

XB and JH designed the experiments. XC and XY carried out the experiments. YS analyzed the experimental results.

## Conflict of Interest Statement

The authors declare that the research was conducted in the absence of any commercial or financial relationships that could be construed as a potential conflict of interest.

## References

[B1] Blazeck J. Garg R. Reed B. Alper H. S. (2012). Controlling promoter strength and regulation in *Saccharomyces cerevisiae* using synthetic hybrid promoters. *Biotechnol. Bioeng.* 109 2884–2895. 10.1002/bit.24552 22565375

[B2] Choi J. W. Da Silva N. A. (2014). Improving polyketide and fatty acid synthesis by engineering of the yeast acetyl-CoA carboxylase. *J. Biotechnol.* 187 56–59. 10.1016/j.jbiotec.2014.07.430 25078432

[B3] Chooi Y. H. Tang Y. (2013). Navigating the fungal polyketide chemical space: from genes to molecules. *ChemInform* 44 9933–9953. 10.1021/jo301592k 22938194 PMC3500441

[B4] Christian H. (2009). The biosynthetic logic of polyketide diversity. *Angew. Chem. Int. Ed.* Engl. 48 4688–4716. 10.1002/anie.200806121 19514004

[B5] Dale S. Wilson W. A. Edelman A. M. Hardie D. G. (1995). Similar substrate recognition motifs for mammalian AMP-activated protein kinase, higher plant HMG-CoA reductase kinase-A, yeast SNF1, and mammalian calmodulin-dependent protein kinase I. *FEBS Lett.* 361 191–195. 10.1016/0014-5793(95)00172-6 7698321

[B6] David F. Nielsen J. Siewers V. (2016). Flux control at the malonyl-CoA node through hierarchical dynamic pathway regulation in *Saccharomyces cerevisiae*. *ACS Synth. Biol.* 5 224–233. 10.1021/acssynbio.5b00161 26750662

[B7] Dixon R. A. Steele C. L. (1999). Flavonoids and isoflavonoids - a gold mine for metabolic engineering. *Trends Plant Sci.* 4 394–400. 10.1016/S1360-1385(99)01471-5 10498963

[B8] Feng X. Lian J. Zhao H. (2015). Metabolic engineering of *Saccharomyces cerevisiae* to improve 1-hexadecanol production. *Metab. Eng.* 27 10–19. 10.1016/j.ymben.2014.10.001 25466225

[B9] Hara T. Sato T. Oka M. Mori S. Shirai H. (2006). Microdiesel: *Escherichia coli* engineered for fuel production. *Microbiology* 152 2529–2536. 10.1099/mic.0.29028-0 16946248

[B10] Hasslacher M. Ivessa A. S. Paltauf F. Kohlwein S. D. (1993). Acetyl-CoA carboxylase from yeast is an essential enzyme and is regulated by factors that control phospholipid metabolism. *J. Biol. Chem.* 268 10946–10952. 8098706

[B11] Hugler M. Menendez C. Schagger H. Fuchs G. (2002). Malonyl-coenzyme a reductase from *Chloroflexus aurantiacus*, a key enzyme of the 3-hydroxypropionate cycle for autotrophic CO_2_ fixation. *J. Bacteriol.* 184 2404–2410. 10.1128/JB.184.9.2404-2410.2002 11948153 PMC134993

[B12] Hunkeler M. Stuttfeld E. Hagmann A. Imseng S. Maier T. (2016). The dynamic organization of fungal acetyl-CoA carboxylase. *Nat. Commun.* 7:11196. 10.1038/ncomms11196 27073141 PMC4833862

[B13] Kawaguchi A. Tomoda H. Nozoe S. Omura S. Okuda S. (1982). Mechanism of action of cerulenin on fatty acid synthetase. Effect of cerulenin on iodoacetamide-induced malonyl-CoA decarboxylase activity. *J. Biochem.* 92 7–12. 10.1093/oxfordjournals.jbchem.a133933 6749834

[B14] Li S. Tong S. Wang M. Zhao H. (2015). Development of a synthetic malonyl-CoA sensor in *Saccharomyces cerevisiae* for intracellular metabolite monitoring and genetic screening. *Acs Synth. Biol.* 4 1308–1315. 10.1021/acssynbio.5b00069 26149896

[B15] Li X. Guo D. Cheng Y. Zhu F. Deng Z. Liu T. (2014). Overproduction of fatty acids in engineered *Saccharomyces cerevisiae*. *Biotechnol. Bioeng.* 111 1841–1852. 10.1002/bit.25239 24752690

[B16] Schujman G. E. Guerin M. Buschiazzo A. Schaeffer F. Llarrull L. I. Reh G. (2006). Structural basis of lipid biosynthesis regulation in Gram-positive bacteria. *EMBO J.* 25 4074–4083. 10.1038/sj.emboj.7601284 16932747 PMC1560364

[B17] Schujman G. E. Paoletti L. Grossman A. D. De Mendoza D. (2003). FapR, a bacterial transcription factor involved in global regulation of membrane lipid biosynthesis. *Dev. Cell* 4 663–672. 10.1016/S1534-5807(03)00123-0 12737802

[B18] Shi S. Chen Y. Siewers V. Nielsen J. (2014). Improving production of malonyl coenzyme A-derived metabolites by abolishing Snf1-dependent regulation of Acc1. *mBio* 5:e1130-14. 10.1128/mBio.01130-14 24803522 PMC4010835

[B20] Wei J. Zhang Y. Yu T. Y. Sadre-Bazzaz K. Rudolph M. J. Amodeo G. A. (2016). A unified molecular mechanism for the regulation of acetyl-CoA carboxylase by phosphorylation. *Cell Discov.* 2:16044. 10.1038/celldisc.2016.44 27990296 PMC5126230

[B21] Woods A. Munday M. R. Scott J. Yang X. Carlson M. Carling D. (1994). Yeast SNF1 is functionally related to mammalian AMP-activated protein kinase and regulates acetyl-CoA carboxylase *in Vivo*. *J. Biol. Chem.* 269 19509–19515. 7913470

[B22] Xu P. Wang W. Li L. Bhan N. Zhang F. Koffas M. A. (2014). Design and kinetic analysis of a hybrid promoter-regulator system for malonyl-CoA sensing in *Escherichia coli*. *ACS Chem. Biol.* 9 451–458. 10.1021/cb400623m 24191643

[B23] Zhang J. Vaga S. Chumnanpuen P. Kumar R. Vemuri G. N. Aebersold R. (2011). Mapping the interaction of Snf1 with TORC1 in *Saccharomyces cerevisiae*. *Mol. Syst. Biol.* 7:545. 10.1038/msb.2011.80 22068328 PMC3261716

