# Supplementary material for: Screening Phosphorylation Site Mutations in Yeast Acetyl-CoA Carboxylase Using Malonyl-CoA Sensor to Improve Malonyl-CoA-Derived Product
Source: Front Microbiol. 2018 Jan 25;9:47. doi: 10.3389/fmicb.2018.00047 (PMC5788913; doi:10.3389/fmicb.2018.00047)
Supplement: Supplementary file 1 [file Data_Sheet_1.docx]

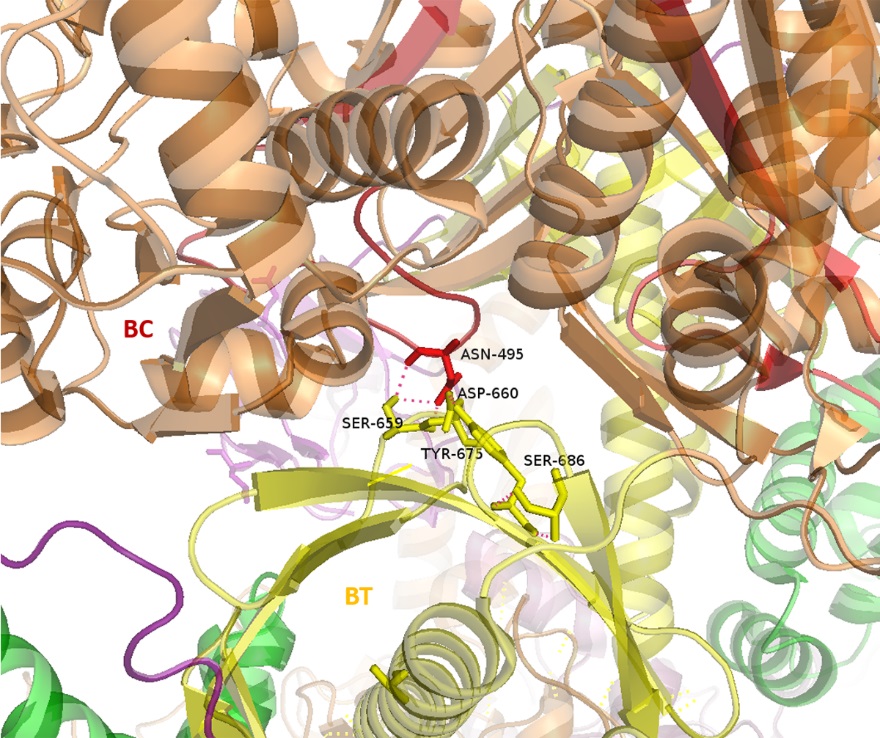


**Figure S1.** Hypothetical structure interaction between BC and BT domain induced by Ser659 and Ser686. The Ser659 residue covalently interacts with the Asn495 residue of the β19β20 loop in the BC domain. The β19β20 loop was demonstrated to bind the biotin motif ([Wei and Tong, 2015](#_ENREF_1)). And the phosphorylation of Ser686 residue in BT domain indirectly regulate the covalent state of Ser659. The structural information of Acc1p was from PDB database and the picture was obtained from PyMOL software.





**Figure S2.** Cell density of Acc1p phosphorylation site mutation containing strains with 3-HP synthesis pathway. Samples were taken every 12h during cultivation in shake flasks. Control(MCR): CEN.PK-102-5B with pIYM plasmid.

**Table S1.** Plasmids used in this study.

| **Plasmid** | **Description** | **Source** |
| --- | --- | --- |
| pYX242WS | 2µm, amp^r^, *TEF1*p-*polyA*, *TPI1*p-*PGK1*t, *LEU2* | Lab store |
| pJfapO | 2µm, amp^r^, *GAL1*(7)*fapO*p-*yeGFP-PGK1*t, *LEU2* | This study |
| pJfapO-fapR | 2µm, amp^r^, *GAL1*(7)*fapO*p-*yeGFP-PGK1*t, *TEF1*p-*fapR*-*polyA*, *LEU2* | This study |
| pIYC04 | 2µm, amp^r^, *TEF1*p-*ADH1*t, *PGK1*p-*CYC1*t, *HIS3* | ([Yun et al. 2013](#_ENREF_1)) |
| pIFapO(0) | 2µm, amp^r^, *fapO*(0)*GAL1*p-*yeGFP-CYC1*t, *HIS3* | This study |
| pIFapO(61) | 2µm, amp^r^, *GAL1*(61)*fapO*p-*yeGFP-CYC1*t, *HIS3* | This study |
| pIFapO(0)-FapR | 2µm, amp^r^, *fapO*(0)*GAL1*p-*yeGFP-CYC1*t, *TEF1*p-*fapR*-nls-*ADH1*t | This study |
| pIFapO(61)-FapR | 2µm, amp^r^, *GAL1*(61)*fapO*p-*yeGFP-CYC1*t, *TEF1*p-*fapR*-nls-*ADH1*t | This study |
| pIYM | pIYC04-*TEF1*p-*MCR*-*ADH1*t, *HIS3* | This study |
| pJFE3 | 2µm, amp^r^, *TEF1*p-*PGK1*t, *URA4* | Lab store |
| pJFE3-fapO | 2µm, amp^r^, *GAL1*(7)*fapO*p -*yeGFP*-*PGK1*t, *URA4* | This study |
| pJS2 | 2µm, amp^r^, *TEF1*p-*ACC1^ser2ala^*-*PGK1*t, *URA4* | This study |
| pJS10 | 2µm, amp^r^, *TEF1*p-*ACC1^ser10ala^*-*PGK1*t, *URA4* | This study |
| pJS60 | 2µm, amp^r^, *TEF1*p-*ACC1*^ser60ala^-*PGK1*t, *URA4* | This study |
| pJT571 | 2µm, amp^r^, *TEF1*p-*ACC1*^thr571ala^-*PGK1*t, *URA4* | This study |
| pJS686 | 2µm, amp^r^, *TEF1*p-*ACC1*^ser686ala^-*PGK1*t, *URA4* | This study |
| pJS882 | 2µm, amp^r^, *TEF1*p-*ACC1*^ser882ala^-*PGK1*t, *URA4* | This study |
| pJS1159 | 2µm, amp^r^, *TEF1*p-*ACC1*^ser1159ala^-*PGK1*t, *URA4* | This study |
| pJS1162 | 2µm, amp^r^, *TEF1*p-*ACC1*^ser1162ala^-*PGK1*t, *URA4* | This study |
| pJS1167 | 2µm, amp^r^, *TEF1*p-*ACC1*^ser1167ala^-*PGK1*t, *URA4* | This study |
| pJS1169 | 2µm, amp^r^, *TEF1*p-*ACC1*^ser1169ala^-*PGK1*t, *URA4* | This study |
| pJT1823 | 2µm, amp^r^, *TEF1*p-*ACC1*^thr1823ala^-*PGK1*t, *URA4* | This study |
| pJS659S1157 | 2µm, amp^r^, *TEF1*p-*ACC1*^ser659alaser1157ala^-*PGK1*t, *URA4* | This study |
| pJS1159S1162 | 2µm, amp^r^, *TEF1*p-*ACC1*^ser1159alaser1162ala^-*PGK1*t, *URA4* | This study |
| pJS1159S1162S1167S1169 | 2µm, amp^r^, *TEF1*p-*ACC1*^ser1159alaser1162alaser1167alaser1169ala^-*PGK1*t, *URA4* | This study |
| pJS686S659S1157 | 2µm, amp^r^, *TEF1*p-*ACC1*^ser686alaser659alaser1157ala^-*PGK1*t, *URA4* | This study |
| pJT1823S659S1157 | 2µm, amp^r^, *TEF1*p-*ACC1*^thr1823alaser659alaser1157ala^-*PGK1*t, *URA4* | This study |

**Table S2.** Strains used in this study.

| **Strain** | **Description** | **Source** |
| --- | --- | --- |
| CEN.PK102-5B | *MATa*; *ura*3-52; *His*3*Δ*1; *leu*2-3,112 | Lab store |
| Control(MCR) | CEN.PK102-5B/pIYM | This study |
| Wild-type | CEN.PK102-5B/pJFE3/ pJfapO-fapR | This study |
| S2A | CEN.PK102-5B/pJS2/pJfapO-fapR | This study |
| S10A | CEN.PK102-5B/pJS10/pJfapO-fapR | This study |
| S60A | CEN.PK102-5B/pJS60/pJfapO-fapR | This study |
| T571A | CEN.PK102-5B/pJT571/pJfapO-fapR | This study |
| S686A | CEN.PK102-5B/pJS686/pJfapO-fapR | This study |
| S882A | CEN.PK102-5B/pJS882/pJfapO-fapR | This study |
| S659S1157A | CEN.PK102-5B/pJS659S1157/pJfapO-fapR | This study |
| S1159A | CEN.PK102-5B/pJS1159/pJfapO-fapR | This study |
| S1162A | CEN.PK102-5B/pJS1162/pJfapO-fapR | This study |
| S1167A | CEN.PK102-5B/pJS1167/pJfapO-fapR | This study |
| S1169A | CEN.PK102-5B/pJS1169/pJfapO-fapR | This study |
| S1159S1162A | CEN.PK102-5B/pJS1159S1162/pJfapO-fapR | This study |
| S1159AS1162AS1167AS1169A | CEN.PK102-5B/pJS1159S1162S1167S1169/ pJfapO-fapR | This study |
| S686AS659AS1157A | CEN.PK102-5B/pJS686S659S1157/pJfapO-fapR | This study |
| T1823A | CEN.PK102-5B/pJT1823/pJfapO-fapR | This study |
| T1823AS659AS1157A | CEN.PK102-5B/pJT1823S659S1157/pJfapO-fapR | This study |
| GAL1p(7)FapO | CEN.PK102-5B/pJfapO | This study |
| GAL1p(7)FapO, HFapR | CEN.PK102-5B/pJfapO-fapR | This study |
| GAL1p(7)FapO, LFapR | CEN.PK102-5B; *TEF1*p*-fapR-ADH1*t, *TEF*p*-KanR-TEF*t, pJFE3-fapO | This study |
| FapO(0)GAL1p | CEN.PK102-5B/pIFapO(0) | This study |
| FapO(61)GAL1p | CEN.PK102-5B/pIFapO(61) | This study |
| FapO(0)GAL1p, HFapR | CEN.PK102-5B/pIFapO(0)-FapR | This study |
| FapO(61)GAL1p, HFapR | CEN.PK102-5B/pIFapO(61)-FapR | This study |
| P-S686A | CEN.PK102-5B/pJS686/pIYM | This study |
| P-T1823A | CEN.PK102-5B/pJT1823/pIYM | This study |
| P-S659AS1157A | CEN.PK102-5B/pJS659S1157/pIYM | This study |
| P-S686AS659AS1157A | CEN.PK102-5B/pJS686S659S1157/pIYM | This study |
| P-T1823AS659AS1157A | CEN.PK102-5B/pJT1823S659S1157/pIYM | This study |

**Table S3.** Primer sequences used in this study.

| Name | Sequence | purpose |
| --- | --- | --- |
| ACC1-F | TTTACTTCTTGCTCATTAGAAAGAAAGCATAGCAATCTAATCTAAGTTTTAATTACAAAGGATCCATGAGCGAAGAAAGCTTATTCGAG | Obtain *ACC1* full length |
| ACC1-R | TGGGGAAAGAGAAAAGAAAAAAATTGATCTATCGATTTCAATTCAATTCAATCCTGCAGGTCGACTTATTTCAAAGTCTTCAACAATTT |  |
| A60-R | CATTATTTGCTATCAGGATCTTAGCTATGACCGTGTGACCACCGTGACTCTTAAC | Mutate serine60 residue to alanine |
| A60-F | GAGTCACGGTGGTCACACGGTCATAGCtAAGATCCTGATAGCAAATAATGGTATTGCC |  |
| A686-R | CAAAGTAGTCATAGAGTCAACAGCTAATCTTGTAGCAGCAACTTCTTCTTTCC | Mutate serine686 residue to alanine |
| A686-F | GAAGAAGTTGCTGCTACAAGATTAgctGTTGACTCTATGACTACTTTGTTGGAAGTTG |  |
| A1823-F | GTAATATGCCAGTTCCTATCTTGGAAGCTAAAGACACATGGGATAGACCAGTTG | Mutate threonine1823 to alanine |
| A1823-R | GGTCTATCCCATGTGTCTTTAGCTTCCAAGATAGGAACTGGCATATTACGC |  |
| A2-F | GAAAGCATAGCAATCTAATCTAAGTTTTAATTACAAAGGATCCATGGCTGAAGAAAGCTTATTCGAGTCTTCTCCACAG | Mutate serine2 to alanine |
| A10-F | GCAATCTAATCTAAGTTTTAATTACAAAGGATCCATGAGCGAAGAAAGCTTATTCGAGTCTGCTCCACAGAAGATGGAGTACGAAATTAC | Mutate serine10 to alanine |
| A571-F | GGACGATTTGATTACTCATAAAATGGCTGCTGAAAAGCCTGATCCAACTCTTG | Mutate threonine571 to alanine |
| A571-R | GAGTTGGATCAGGCTTTTCAGCAGCCATTTTATGAGTAATCAAATCGTCCAACCAAC |  |
| A659-F | TAAATGTGATATCATACTGCGTCAACTAGCTGATGGTGGTCTTTTGATTGCCATAGGCG | Mutate serine659 to alanine |
| A659-R | CGCCTATGGCAATCAAAAGACCACCATCAGCTAGTTGACGCAGTATGATATCACATTTA |  |
| A1157-F | AAATCTAAAATGGGTATGAACAGGGCTGTTGCTGTTTCAGATTTGTCATATGTTGCAAA | Mutate serine1157 to alanine |
| A1157-R | TGAAACAGCAACAGCCCTGTTCATACCCATTTTAGATTT |  |
| A1169-F | CATATGTTGCAAACAGTCAGGCTTCTCCGTTAAGAGAAGGTATTTTGATGGCT | Mutate serine1169 to alanine |
| A1169-R | TACCTTCTCTTAACGGAGAAGCCTGACTGTTTGCAACATATGACAAATCTG |  |
| A882-F | GGTGCTGTTTTCCCAGCTAGACAATTAGCTAAATTGATTGATATGGCCGTGAAGAATCCT | Mutate serine882 to alanine |
| A882-R | CGGCCATATCAATCAATTTAGCTAATTGTCTAGCTGGGAAAACAGCACC |  |
| A1162-F | TGTTTCTGTTTCAGATTTGGCTTATGTTGCAAACAGTCAGTCATC | Mutate serine1162 to alanine |
| A1162-R | CTGACTGTTTGCAACATAAGCCAAATCTGAAACAGAAACAGCC |  |
| A1167-F | TTTGTCATATGTTGCAAACGCTCAGTCATCTCCGTTAAGAGAAG | Mutate serine1167 to alanine |
| A1167-R | TCTTAACGGAGATGACTGAGCGTTTGCAACATATGACAAATCTG |  |
| A1159-F | GTATGAACAGGGCTGTTTCTGTTGCTGATTTGTCATATGTTGCAAACAGTCAGTC | Mutate serine1159 to alanine |
| A1159-R | CTGTTTGCAACATATGACAAATCAGCAACAGAAACAGCCCTGTTCATACC |  |
| AD-F | TGAACAGGGCTGTTTCTGTTGCTGATTTGGCTTATGTTGCAAACAGTCAGTCATCTCCG | Mutate serine1159, serine1162, serine1167, serine1169 to alanine |
| AD-R | CTGACTGTTTGCAACATAAGCCAAATCAGCAACAGAAACAGCCCTGTTCATACC |  |
| AF-F | GTATGAACAGGGCTGTTTCTGTTGCTGATTTGGCTTATGTTGCAAACGCTCAGGCTTCTCCGTTAAGAGAAGGTATTTTGATGGC |  |
| AF-R | CCTTCTCTTAACGGAGAAGCCTGAGCGTTTGCAACATAAGCCAAATCAGCAACAGAAACAGCCCTGTTCATACC |  |
| FapR-F | CAAGTCGACATGAGAAGAAATAAAAGAGAAAGGC | Construct plasmids pJfapO and pJfapO-FapR |
| FapR-R | CGAGCTCGTTAAGAATGTTTAGATCTATACATATCG |  |
| TEF1-F | GGTTCCGCGCACATTTCCCCGAAAAGTGCCACCTGACGTCATAGCTTCAAAATGTTTCTACTCCT |  |
| TEF1-FapO-R | GTTATGCAGCTTTTCCATTTATATATTAGTACCTGATACTAAGCCTTTTTCGACGAAGAAAAAG |  |
| GAL1core-F | TATATAAATGGAAAAGCTGCATAACCACTTTAACT |  |
| GAL1-R | CCCGGGTATAGTTTTTTCTCCTTGACGTTAAAG |  |
| GFP-F | CTTTAACGTCAAGGAGAAAAAACTATACCCGGGATGTCTAAAGGTGAAGAATTATTCAC |  |
| GFP-R | GACATGGGAGATCCTAGCTAGCTAGATCCATGGTGAATTCTTATTTGTACAATTCATCCATACCATGG |  |
| PI-FapR-F | ATTTGCGGCCGCTTTAATGAGAAGAAATAAAAGAGAAAGGC | Construct plasmids pIFapO(0), pIFapO(61), pIFapO(0)-FapR and pIFapO(61)-FapR |
| PI-FapR-nls-R | CGAGCTCGTTAAACCTTTCTCTTCTTCTTTGGAGAATGTTTAGATCTATACATATCG |  |
| TEF1-FapO(0)-R | GTTATGCAGCTTTTCCATTTATATATTAGTACCTGATACTAAGCCTTTTTCGACGAAGAAAAAG |  |
| GFP-SbfI-R | CCTGCAGGTTATTTGTACAATTCATCCATACCATGG |  |
| FapO(61)-F | AGTTTGTATTACTTAGTATCAGGTACTAATTCTTATTCAAATGTCATAAAAGTATC |  |
| FapO(61)-R | TTAGTACCTGATACTAAGTAATACAAACTGAAAATGTTGAA |  |
| RK1-F | CGGCAGAGACCAATCAGTAAAAATC | Integrate *fapR* to genome |
| RK1-R | ATTTTGAAGCTATGGTGTGTGCGGCCTTTGTGTGCTTAATCACGTATAC |  |
| RK2-F | GGTGATATCAGATCCACTAGTGGCTAAGTATTGTTTGTGCACTTGCCTG |  |
| RK2-R | GAAAATGATTACGAGGATACGGAGAG |  |
| FapR1 -F | CGCACACACCATAGCTTCAAAAT |  |
| FapR1 -R | ATTAAGGGTTGTCGACCTGCAGTTCGCTATTACGCCAGCTGG |  |
| G418-F | CTGCAGGTCGACAACCCTTAAT |  |
| G418-R | GCCACTAGTGGATCTGATATCACC |  |

**Condon optimized sequence of *MCR***

ATGTCAGGTACTGGTAGATTGGCAGGAAAAATAGCTTTAATAACTGGTGGTGCAGGTAATATTGGTTCTGAATTGACTAGGAGGTTCTTGGCAGAAGGTGCAACTGTTATTATATCTGGTAGAAACAGAGCAAAGTTAACTGCTTTAGCTGAAAGGATGCAAGCAGAGGCTGGAGTTCCTGCTAAGAGGATTGACTTGGAAGTCATGGATGGATCAGATCCTGTCGCTGTTAGGGCTGGTATTGAGGCTATAGTCGCTAGGCACGGTCAAATTGACATTTTAGTTAACAACGCAGGATCTGCTGGTGCTCAAAGAAGATTGGCTGAAATTCCATTAACAGAAGCTGAGTTGGGTCCTGGAGCTGAAGAAACATTACATGCATCAATTGCTAATTTATTGGGTATGGGTTGGCACTTGATGAGAATTGCAGCTCCTCACATGCCTGTTGGTTCTGCAGTTATTAACGTTTCTACTATTTTTTCTAGGGCTGAATACTACGGTAGGATTCCATACGTCACTCCAAAGGCAGCATTGAATGCTTTATCTCAATTAGCTGCTAGAGAATTGGGAGCTAGAGGTATAAGAGTTAACACTATATTCCCAGGTCCAATTGAGTCTGATAGAATAAGAACAGTTTTTCAAAGAATGGATCAGTTGAAGGGTAGACCTGAGGGTGATACTGCTCATCATTTCTTAAACACTATGAGGTTGTGCAGAGCAAACGATCAGGGTGCATTGGAGAGAAGATTCCCATCAGTCGGAGATGTTGCTGACGCTGCTGTTTTCTTGGCTTCTGCAGAATCTGCTGCATTGTCTGGTGAGACTATAGAAGTTACACATGGAATGGAGTTGCCAGCATGCTCAGAGACTTCTTTATTAGCTAGAACAGATTTGAGAACAATTGACGCTTCAGGAAGGACAACTTTAATTTGTGCTGGAGATCAGATTGAAGAGGTCATGGCTTTGACAGGTATGTTGAGGACATGTGGTTCAGAAGTTATTATTGGTTTTAGGTCAGCTGCTGCTTTGGCACAGTTTGAACAAGCAGTCAATGAGTCTAGGAGGTTGGCAGGTGCTGACTTTACACCACCTATTGCATTGCCTTTGGACCCAAGGGACCCTGCTACAATTGATGCTGTCTTCGATTGGGGTGCAGGTGAAAATACAGGTGGTATACACGCAGCAGTTATTTTACCAGCTACTTCTCATGAGCCAGCTCCTTGCGTCATAGAGGTTGATGATGAAAGAGTCTTGAATTTCTTGGCAGATGAGATTACAGGTACAATTGTCATTGCATCAAGATTGGCTAGGTACTGGCAGTCACAGAGGTTAACACCTGGTGCAAGGGCTAGAGGTCCAAGAGTCATCTTTTTATCTAACGGTGCTGACCAAAATGGTAACGTCTATGGTAGAATTCAATCTGCTGCTATTGGTCAATTGATAAGAGTCTGGAGGCACGAAGCTGAATTGGACTACCAGAGAGCATCTGCAGCTGGTGATCATGTCTTGCCTCCAGTCTGGGCAAACCAAATTGTCAGGTTCGCAAACAGGTCATTGGAGGGTTTGGAATTCGCATGCGCTTGGACAGCACAGTTATTACATTCTCAAAGACACATAAACGAAATTACTTTAAATATTCCAGCTAATATTTCTGCAACAACTGGTGCTAGGTCTGCTTCAGTCGGTTGGGCTGAATCATTAATTGGTTTACATTTGGGTAAAGTTGCTTTGATTACTGGTGGTTCAGCTGGAATAGGAGGTCAGATAGGTAGGTTGTTGGCTTTGTCTGGAGCTAGAGTCATGTTAGCTGCTAGGGACAGACACAAATTAGAACAAATGCAAGCTATGATACAATCTGAATTAGCTGAAGTTGGTTACACTGATGTCGAGGACAGGGTTCATATTGCTCCAGGTTGCGACGTCTCATCTGAAGCTCAATTGGCAGATTTAGTTGAAAGGACATTGTCTGCTTTTGGTACAGTCGATTATTTGATTAATAACGCTGGAATAGCTGGTGTCGAGGAAATGGTCATAGACATGCCTGTCGAAGGATGGAGGCATACATTGTTCGCAAACTTAATTTCTAACTATTCTTTGATGAGAAAATTGGCACCATTGATGAAGAAGCAAGGTTCTGGATACATTTTGAATGTCTCTTCTTACTTTGGTGGTGAGAAGGACGCAGCTATTCCTTACCCAAACAGGGCTGACTACGCAGTCTCAAAAGCTGGACAGAGAGCTATGGCTGAGGTTTTTGCTAGGTTCTTGGGTCCTGAAATTCAAATTAATGCTATAGCACCAGGTCCAGTCGAAGGAGATAGGTTGAGAGGTACTGGTGAGAGGCCTGGTTTGTTCGCTAGAAGAGCTAGATTAATTTTAGAAAATAAGAGATTGAATGAGTTGCACGCAGCATTGATTGCTGCTGCTAGGACTGACGAAAGGTCAATGCACGAATTGGTTGAATTGTTATTGCCTAACGACGTCGCTGCATTAGAGCAGAATCCAGCTGCTCCTACTGCTTTAAGGGAGTTGGCTAGGAGGTTTAGATCAGAGGGTGACCCAGCAGCTTCTTCATCTTCTGCATTGTTGAATAGATCAATAGCAGCTAAGTTATTAGCAAGATTGCATAATGGTGGTTATGTTTTGCCAGCTGACATATTTGCAAACTTGCCAAACCCTCCTGACCCATTCTTCACAAGGGCACAAATTGATAGGGAGGCTAGGAAGGTTAGGGATGGAATTATGGGTATGTTATATTTGCAGAGGATGCCAACAGAGTTCGACGTCGCTATGGCTACTGTCTATTATTTGGCTGACAGAAACGTCTCAGGAGAGACATTTCATCCATCAGGTGGTTTAAGATATGAAAGAACACCAACTGGTGGTGAGTTGTTCGGTTTGCCATCTCCTGAGAGATTGGCAGAGTTAGTTGGTTCTACAGTTTACTTAATTGGTGAACATTTAACTGAACATTTAAATTTGTTAGCTAGGGCTTACTTGGAAAGGTACGGAGCTAGACAAGTTGTTATGATTGTCGAAACTGAAACTGGTGCAGAGACTATGAGGAGGTTATTGCACGACCACGTTGAAGCTGGTAGGTTGATGACAATTGTCGCAGGTGACCAGATTGAAGCTGCAATAGACCAAGCTATAACAAGATATGGTAGACCAGGACCAGTCGTCTGTACTCCATTCAGGCCATTACCAACTGTCCCTTTGGTTGGTAGAAAAGACTCTGATTGGTCAACTGTTTTATCTGAAGCAGAATTCGCAGAGTTGTGCGAGCATCAATTGACTCATCATTTTAGAGTTGCTAGGAAGATTGCTTTATCTGACGGTGCTTCATTGGCTTTGGTCACTCCAGAGACAACTGCAACATCAACTACTGAGCAATTCGCTTTGGCAAACTTTATTAAAACTACTTTACACGCTTTCACTGCTACTATTGGAGTTGAGTCTGAAAGAACTGCTCAAAGGATTTTGATTAATCAAGTTGACTTGACTAGAAGAGCTAGGGCTGAAGAGCCAAGAGATCCTCACGAGAGACAACAGGAGTTGGAAAGATTTATAGAGGCAGTTTTATTGGTTACTGCTCCATTACCTCCTGAGGCTGACACAAGGTACGCTGGTAGGATACATAGGGGTAGGGCAATTACTGTTTAA

**Condon optimized sequence of *fapR***

ATGAGAAGAAATAAAAGAGAAAGGCAAGAATTGTTGCAACAAACTATTCAAGCTACTCCATTCATTACTGATGAAGAATTAGCTGGTAAATTTGGTGTTTCTATTCAAACTATTAGATTAGATAGATTGGAATTGTCTATTCCAGAATTGAGAGAAAGAATTAAAAATGTTGCTGAAAAAACTTTGGAAGATGAAGTTAAATCTTTGTCTTTAGATGAAGTTATTGGTGAAATTATTGATTTGGAATTGGACGATCAAGCTATTTCTATTTTGGAAATTAAGCAAGAACATGTTTTTTCAAGAAATCAAATTGCTAGAGGTCACCACTTGTTCGCACAGGCTAACTCTTTGGCTGTCGCTGTCATTGACGACGAATTGGCTTTGACAGCTTCTGCTGACATTAGATTTACTAGACAGGTCAAGCAAGGTGAAAGGGTCGTTGCAAAGGCAAAGGTCACTGCTGTCGAGAAGGAGAAGGGTAGAACTGTCGTCGAGGTCAACTCTTACGTCGGTGAAGAAATTGTTTTTTCTGGTAGATTCGATATGTATAGATCTAAACATTCTTAA
